# Sanguineous Effusion behind the Uterus

**Published:** 1851-08

**Authors:** 


*Sanguineous effusion behind the Uterus.*—At a late meeting of the Surgical Society of Paris, M. Monod related the following case:—A woman was admitted into the Maison de Santé with severe pain in the pelvic region. This case was first looked upon as one of retroversion of the uterus, but it was soon discovered that a considerable fluctuating tumour existed just behind the womb. Two punctures were made at a little interval, which both yielded blood; the pain, however, did not diminish, and the patient died. On a post-mortem examination, a large sac was found behind the uterus, and close upon that organ; it was divided into two distinct halves, both filled with blood.—*London Lancet, July* 5, 1851.

